# Clonal diversity and epidemiological characteristics of *Staphylococcus aureus*: high prevalence of oxacillin-susceptible *mec*A-positive *Staphylococcus aureus* (OS-MRSA) associated with clinical isolates in Brazil

**DOI:** 10.1186/s12866-016-0733-4

**Published:** 2016-06-21

**Authors:** Mariana Andrade-Figueiredo, Tereza Cristina Leal-Balbino

**Affiliations:** Department of Microbiology, Oswaldo Cruz Foundation, Aggeu Magalhães Research Center, CPqAM/Fiocruz, Av. Professor Moraes Rego, s/n - CamLpus da UFPE - Cidade Universitária, Recife, PE 50.670-420 Brazil

**Keywords:** *Staphylococcus aureus*, MRSA, MSSA, OS-MRSA, Genotyping

## Abstract

**Background:**

*Staphylococcus aureus* is the major cause of global and nosocomial infections with a significant impact in hospitals worldwide. Our objective was to investigate clinical and molecular characteristics of *S. aureus* isolates causing infections in patients admitted to hospitals from Recife city, Brazil, and investigate the prevalence of oxacillin-susceptible *mec*A-positive *S. aureus* (OS-MRSA) in the region, as well as genetically characterize the isolates and compare with epidemic clones.

**Results:**

We characterized 89 isolates in total, 31 clinical methicillin-resistant *S. aureus* (MRSA) and 58 methicillin-sensitive (MSSA) isolates by PFGE, MLST, *spa* typing and SCC*mec* genotyping. Isolates belonging to international MRSA clones were present: Brazilian epidemic clone (BEC) (61 % of MRSA isolates), Paediatric (36 %), New York/Japan (3 %). Some MSSA isolates were related to MRSA clones: USA400-related (10 % of MSSA isolates), Berlin clone (2 %), Paediatric (14 %), New York/Japan (2 %) and Southwest Pacific clone (17 %). MLST revealed new sequence types (ST’s): ST2381, ST2382, and ST2383 and new *spa* types: 10548 and 10550. Among isolates phenotypically identified as MSSA by antimicrobial susceptibility assays, we verified 30 oxacillin-susceptible isolates, which exhibited the *mec*A gene, without *mec* complex amplification and were thus classified as OS-MRSA. We observed clonal spread of MRSA and MSSA, including OS-MRSA, within several areas of the main hospital investigated and closely related isolates between hospitals analyzed.

**Conclusions:**

The results of this study suggest a possible spread of the strains in hospital environment that could be responsible for nosocomial infections. We documented the presence of several MRSA clones, as well as new MLST and *spa* types, that were responsible for severe infections in hospitalized patients. The finding of OS-MRSA isolates could have implications for therapy, because testing for *mec*A and PBP2a is not a routine procedure performed by clinical microbiology laboratories in Brazil and, as consequence, these isolates could be misclassified as MSSA. Our data alert to the necessity to develop more effective strategies for epidemiological control of *S. aureus* in order to avoid an increase of hospital infections provoked by this pathogen. We reinforce the use of genetic methods, in addition to phenotypic tests, for a precise identification of MRSA.

**Electronic supplementary material:**

The online version of this article (doi:10.1186/s12866-016-0733-4) contains supplementary material, which is available to authorized users.

## Background

Methicillin-resistant *Staphylococcus aureus* (MRSA) has become widespread in hospitals, causing both serious nosocomial and community-associated infections worldwide. However, methicillin-sensitive *S. aureus* (MSSA), which generally is genetically more diverse than MRSA, remains an important cause of infection [1, 2].

Methicillin-resistance in *S. aureus* results from acquisition of the *mec*A gene, harbored on a mobile genetic element (MGE), staphylococcal cassette chromosome (SCC*mec*), which produces an alternative penicillin-binding protein (PBP2a) with a low affinity for β-lactam antibiotics [2, 3]. Hospital-associated (HA)-MRSA isolates usually carry SCC*mec*I, II, or III, whereas community-associated (CA)-MRSA strains commonly carry SCC*mec*IV, and less frequently, SCC*mec*V or VII [3, 4]. MRSA has been defined as *S. aureus* having the *mec*A gene or phenotypically showing resistance to oxacillin/cefoxitin. However, some isolates carry the *mec*A gene but are susceptible to oxacillin/cefoxitin, referred to as oxacillin-susceptible MRSA (OS-MRSA), also known as cefoxitin-sensitive MRSA, which have been reported worldwide [5–8].

MRSA accounted for 54 % of nosocomial *S. aureus* infections in Brazil in 2006 [9]. A multiresistant HA-MRSA clone, known as Brazilian epidemic clone (BEC), characterized as sequence type (ST) 239 and SCC*mec*III, is responsible for the majority of nosocomial infections in Brazil [10–12]. However, more recent studies reported a frequent occurrence of the MRSA USA800/Paediatric clone (PC) (ST5-SCC*mec*IV), USA100/New York/Japan clone (ST5-SCC*mec*II), USA400 (ST1-SCC*mec*IV) and USA1100/Southwest pacific clone (SWP) (ST30-SCC*mec*IV) in Brazilian hospitals [13–18].

The aim of this study was to investigate clinical and molecular characteristics of *S. aureus* isolates that cause infections in patients admitted to hospitals from Recife city, Brazil, and investigate the prevalence of oxacillin-susceptible *mec*A-positive *S. aureus* (OS-MRSA) in the region, as well as genetically characterize the isolates and compare with epidemic clones. Little is known about the prevalence of these isolates causing infections in patients admitted to hospitals in the country, especially in Northeast region. Therefore, the results may be helpful to warn committees of nosocomial infection control about the persistence and introduction of epidemic clones into hospitals and to alert for the development of more efficient control strategies to reduce hospital infection.

## Methods

### Ethics statement

The project was approved by the Oswaldo Cruz Foundation Health Research Ethics Committee, Aggeu Magalhães Research Center, CPqAM/Fiocruz, Brazil (CEP: 0024.0.095.000-07) and the University of Pittsburgh Institutional Review Board (PRO11030330). The present study involved use of existing *S. aureus* isolates obtained from the microbiology laboratory of each hospital. The samples were obtained from the routine clinical care. There was no contact with human subjects and no access to personal patient information. Therefore, no informed consent was obtained for this study. This consent procedure was approved by both ethics committees.

### Setting and selection of bacterial isolates

The study was conducted in Recife, a city of approximately 1.5 million inhabitants located in Pernambuco State in Northeast Brazil and the experiments were performed in the Infectious Diseases Epidemiology Research Unit of the University of Pittsburgh, USA. A total of 89 isolates of *S. aureus* were obtained from clinical specimens of patients from outpatient clinics, inpatient wards and intensive care units (ICU) of hospitals in Recife that provide care to patients from different regions of Pernambuco, obtained from spontaneous demand. We collected all isolates identified as *Staphylococcus aureus* in the microbiology laboratories and that were responsible for infection in patients admitted into the hospitals. Eighty isolates (Sa1-Sa80) were from a general university hospital (hospital 1), collected during 2009; Four isolates (Sa82, Sa86, Sa87, Sa89) were obtained from a second general university hospital (hospital 2) and the remaining five isolates (Sa81, Sa83-Sa85, Sa88) were obtained from a cardiology hospital (hospital 3), all samples collected in 2011. A single isolate was obtained from each patient and reconfirmed as *S. aureus* by coagulase and mannitol fermentation tests, as well as by PCR of the coagulase gene (*coa*) [19]. As exclusion criteria of the study, were not considered for analysis isolates not identified as *Staphylococcus aureus* in the microbiology laboratories of the hospitals, isolates without informations of the source of infection and the area where the patient was admitted.

### Phenotypic identification of MRSA

Antimicrobial susceptibility testing was performed by disc diffusion method on Muller-Hinton agar (BD-Becton, Dickinson and Company, Franklin Lakes, NJ) according to the recommendations of the Clinical and Laboratory Standards Institute (CLSI) [20] with the antibiotic cefoxitin (30 μg). The plates were incubated at 35 °C with an initial reading after incubation of 24 h, and a second reading after incubation of 48 h. The Minimum inhibitory concentrations (MICs) of cefoxitin were determined using an E-test (bioMérieux, Lyon, France) and using an agar dilution method with cefoxitin as preconized by CLSI [20]. Representatives of all clusters and all sporadic oxacillin-susceptible *mec*A-positive (OS-MRSA) isolates were passaged 5 times on Muller-Hinton agar plates with subinhibitory concentrations of cefoxitin (0.5 μg/ml) and then cefoxitin MICs were determined using E-test (bioMérieux, Lyon, France). The same methodology was used for the isolates before and after passages. *S. aureus* ATCC 33591 and ATCC 25923 were included as quality control strains.

### Molecular characterization

Genomic DNA was extracted using the automated NucliSens-easyMAG (bioMérieux, Durham, NC). PCR was performed on a GeneAmp PCR System 9700 (Applied Biosystems, Foster City, CA) and Sanger sequencing of gene loci was performed using the Big Dye Terminator Kit v3.1 and an ABI 3730xl DNA analyzer (Applied Biosystems, Foster City, CA).

#### SCC*mec* typing

To determine if the isolates harbored segments of SCC*mec* elements I to V, identification of the SCC*mec* complex was performed using two multiplex PCRs [21]; MPCR-1, that identifies five types of *ccr* genes (*ccrAB1*[1], *ccrAB2*[2], *ccrAB3*[3], *ccrAB4*[4], and *ccrC*[5]), in which amplification of the *mec*A gene was used as an internal control; and MPCR-2 that identifies class A to class C of *mec* complex [21]. The following *S. aureus* strains were used as positive controls: MRSA NCTC10442 (SCC*mec*I, class B *mec*, c*crAB1*), MRSA N315 (SCC*mec*II, class A *mec, ccrAB2*), MRSA 85/2082 (SCC*mec*III, class A *mec*, *ccrAB3*), MRSA WIS (SCC*mec*V, class C *mec*, *ccrC*) [21] and MRSA1 (SCC*mec*IV, class B *mec*, *ccrAB2*) [22].

#### MLST, *spa* typing, and PFGE

Multilocus sequence type (MLST) was performed as previously described [23]. Analysis of chromatograms and sequences was performed using Lasergene’s SeqMan Pro package (version 10.0.1, DNAStar, Madison WI). MLST sequences obtained were submitted to http://saureus.mlst.net for generation of allelic profiles and to assign the sequence type (ST). STs were assigned to a clonal complex (CC) using eBURST (Based Upon Related Sequence Types) algorithm analysis (http://eburst.mlst.net/). A CC was defined as having at least six of seven identical loci [24].

*spa* typing was performed as previously described [25] and *spa* types were determined using Ridom StaphType (version 1.5.21, Ridom GmbH, Würzburg, Germany) and the Ridom SpaServer (http://spa.ridom.de/). Using the Based Upon Repeat Pattern (BURP) algorithm within Ridom Staphtype software, *spa* types were also grouped into *spa* clonal complexes (*spa*CC). BURP analysis allows determination of clonal relatedness based on *spa* types of *S. aureus* [26]. *S. aureus* strains MRSA1 (ST1, t316), MRSA WIS (ST45, t123), MRSA 85/2082 (ST239, t037), MRSA N315 (ST5, t002), MRSA NCTC10442 (ST250, t008) were used as positive controls.

Pulsed-field gel electrophoresis (PFGE) was performed as previously described [22] on a CHEF-DR III SYSTEM (Bio-Rad, Hercules, CA), using *SmaI* enzyme (30 units per sample) [27]. The PFGE patterns were analyzed using BioNumerics software (version 6.5, Applied Maths, Austin, TX) and isolates were grouped into pulsed-field types using UPGMA, >80 % relatedness with 1.5 % of similarity tolerance and 1.5 % of Dice optimization. The PFGE profiles were also analyzed based on the criteria of Tenover and coworkers [27]. The clusters were compared with the pulsed-field patterns USA100, USA300, USA1000, USA500, USA900, USA400, USA600, USA700, USA800, USA1100 and USA1200 [22]. The reference standard *S. aureus* NRS77 was used as the global-standard *S. aureus*.

## Results

### Genotyping and epidemiological characteristics

#### *Staphylococcus aureus* methicillin resistance (MRSA) isolates

A total of 31/89 (35 %) *S. aureus* were considered to be MRSA by both antimicrobial susceptibility assays (cefoxitin disc diffusion range to 6–12 mm of diameter and cefoxitin MICs range of >4–128 μg/mL) and *mec*A gene detection. The SCC*mec* typing for MRSA isolates showed that 19/31 (61 %) isolates were SCC*mec* type III, 11/31 (36 %) isolates were SCC*mec* type IV and a single isolate was SCC*mec* type II.

**Fig. 1:**
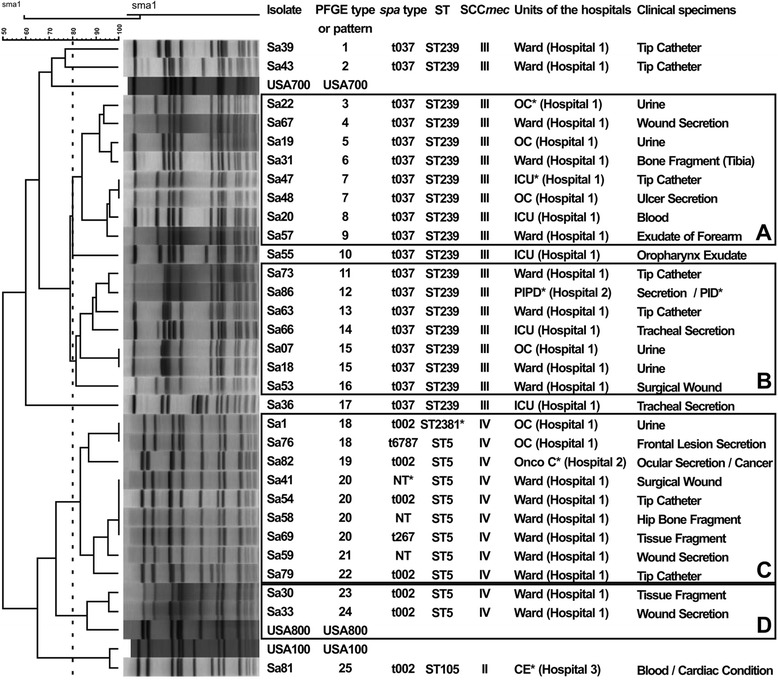
PFGE dendrogram of 31 MRSA isolates and reference strains. OC* = Outpatient Clinic; ICU* = Intensive Care Units; OncoC* = Oncology Center; CE* = Cardiology Emergency; NT* = nontypeable; PIPD* = Pavilion of Infectious and Parasitic Diseases; PID* = Parasitic/Infectious Disease; ST2381* = new ST described in this study

**Fig. 2:**
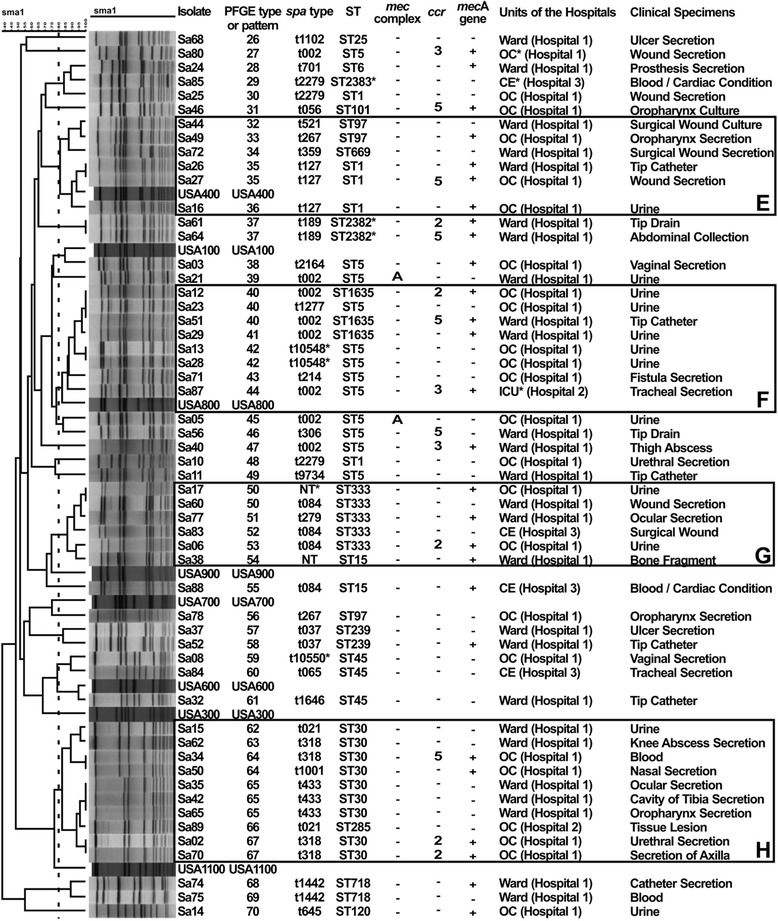
PFGE dendrogram of 55 MSSA isolates and reference strains. OC* = Outpatient Clinic; ICU* = Intensive Care Units; CE* = Cardiology Emergency; NT* = nontypeable; ST2382* and ST2383* = new ST’s described in this study; t10548* and t10550* = new *spa* types described in this study. Isolates that are *mec*A gene positive were considered to be oxacillin-susceptible, *mec*A-positive *S. aureus* (OS-MRSA). Three MSSA isolates determined to be OS-MRSA (Sa4, Sa9, Sa45) are not shown because they were nontypeable by PFGE

PFGE analysis revealed four predominant clusters each for MRSA and MSSA isolates, designated as A-D (Fig. 1) and E-H (Fig. 2), respectively. Some clusters included several of the major international MRSA clones (Clusters A/B/C included BEC; Cluster D and F, USA800/PC; Cluster E, USA400). Only a few isolates not included in these clusters were individually related to epidemic clones (isolates Sa3 [USA100/New York/Japan] and Sa32 [USA600/Berlin clone], Fig. 2, and Sa81 [USA100], Fig. 1).

All MRSA isolates belonging to SCC*mec* type III were classified as ST239 and exhibited *spa* type t037, being therefore related to the BEC clone (clusters A and B, Fig. 1). All five MRSA isolates from ICU patients in hospital 1 were classified as related to BEC (Fig. 1, Sa47, 20, 55, 66, 36).

The 11 MRSA isolates belonging to SCC*mec* type IV (Fig. 1, clusters C and D) were related to USA800/PC, having SCC*mec*IV-ST5 and a PFGE pattern similar to USA800 according to Deurenberg and Stobberingh [28]. Despite the PFGE pattern USA800 could not being grouped into cluster C, the isolates (SCC*mec*IV-ST5, except Sa1 SCC*mec*IV-ST2381) were most closely related to the Pediatric Clone (USA800/PC), exhibiting no more than 3 PFGE band difference from the USA800 pattern by Tenover criteria [27]. Isolate Sa1 was classified as *spa* type t002 and had a new MLST allele at *tpi* (*tpi*- 264), being designated as ST2381, which belongs to CC5. The amplification of *spa* failed for 3 isolates (Sa41, Sa58 and Sa59) and the isolates were considered as *spa* nontypeable. Isolate Sa81 from the blood of a cardiology patient from hospital 3 was related to the New York/Japan clone (USA100) and was classified as SCC*mec* type II, ST105/t002 (Fig. 1), belonged to the same clonal complex of ST5 isolates (CC5), when analyzed by the BURST algorithm.

#### *Staphylococcus aureus* methicillin susceptible (MSSA) isolates

Among the 58 isolates phenotypically identified as MSSA by antimicrobial susceptibility assays (cefoxitin disc diffusion range to 25–30 mm of diameter and cefoxitin MICs range of ≤ 4 μg/mL), there were two new ST’s (ST2382 and ST2383, Fig. 2). Isolates Sa4 and Sa8 had a previously unknown *spa* type, designated as t10550, which is similar to *spa* type t938. Two other isolates (Sa13 and Sa28, both ST5), exhibited a previously-unknown *spa* type, classified as t10548, which is similar to *spa* type t5344. Isolates Sa17 and Sa38 amplified the *spa* gene but sequencing was unsuccessful.

The MSSA isolates related to USA400 were grouped into cluster E. Two ST5 isolates (*spa* type t10548) and 3 ST1635 isolates (t002) from cluster F were related to the USA800/PC, additionally, isolate Sa87 (t002) from an ICU patient in hospital 2, Sa23 (t1277) and Sa71 (t214) from outpatient clinic of hospital 1 also belonged to the USA800/PC.

The isolates that exhibited ST333 (Sa17, Sa60, Sa77, Sa83 and Sa06) and one isolate ST 15 (Sa38) were grouped into cluster G. All the ST30 isolates and a singleton ST285 isolate were grouped into cluster H. Despite the PFGE pattern USA1100 could not being grouped into cluster H, the isolates were most closely related to the Southwest Pacific clone (SPW, USA1100), exhibiting no more than 3 PFGE band difference from the USA1100 profile by Tenover criteria [27] (Fig. 2). Two MSSA isolates were individually related to epidemic clones. Isolate Sa03 (ST5/t2164) was related to the USA100/New York/Japan clone and Sa32 was related to the USA600/Berlin clone (ST45-SCC*mec*IV).

### Oxacillin-susceptible *mec*A-positive *S. aureus* isolates

The 58 isolates, considered MSSA by both antimicrobial susceptibility assays, were investigated to determine if they harbored segments of SCC*mec*. Twenty five (43 %) were negative for all SCC*mec* genes investigated. However, 30 (52 %) isolates, cefoxitin MICs in the range of 2–4 μg/mL, were *mec*A gene positive, of which 15 isolates also amplified *ccr* genes types 2, 3 or 5, without *mec* complex amplication (Fig. 2). These isolates were thus classified as oxacillin-susceptible *mec*A-positive *S. aureus* (OS-MRSA) and were highly diverse by MLST and PFGE (Fig. 2). In order to confirm if OS-MRSA isolates observed can be truly oxacillin-susceptible, representatives of all clusters and all sporadic oxacillin-susceptible *mec*A-positive isolates (Sa3, Sa14, Sa24, Sa26, Sa34, Sa40, Sa46, Sa52, Sa61, Sa64, Sa74, Sa77, Sa80, Sa87 and Sa88) were tested with subinhibitory concentrations of cefoxitin and cefoxitin MICs were determined. As a result, cefoxitin MICs, before passages, range of 1.5–4.0 μg/mL. Additionally, cefoxitin MICs, after passages, range of 1.5–4.0 μg/mL and therefore, all isolates tested were considered susceptible to cefoxitin.

Isolates Sa49, Sa26, Sa27 and Sa16 were grouped into cluster E and related to USA400. The isolates Sa61 and Sa64 exhibited the new ST2382 described. The OS-MRSA isolate Sa03 was related to USA100/New York/Japan. Isolates Sa12, Sa51, Sa29 and Sa87 were grouped into cluster F and related to USA800/PC. The OS-MRSA isolates Sa17, Sa77, Sa06 (ST333) and Sa38 (ST15) were grouped into cluster G. Isolate Sa52 exhibited ST239 and t037, similar to BEC clone and the isolates Sa34, Sa50, Sa02 and Sa70 (ST30) were grouped into cluster H (Fig. 2). Isolates Sa14 and Sa74 exhibited a faint PCR product band for *ccr* genes and were not considered for the analysis. The remaining 3 isolates were *mec*A negative, although they harbored *mec* complex type A (Sa5 and Sa21) and *ccr* gene type 5 (Sa56) (Fig. 2).

We observed clonal spread of MRSA and MSSA, including OS-MRSA isolates, within the main hospital analyzed (hospital 1). We also observed closely related isolates between hospital 1 and all four isolates from hospital 2 (Figs. 1 and 2), as well as hospital 3 (Sa83, 84, 85) (Fig. 2). Only 3 isolates (OS-MRSA) were untypeable by PFGE, Sa4 (ST-nontypeable/t10550), Sa9 (ST-nontypeable/t037) and Sa45 (ST398/t1451). These results were confirmed by repeating running (PFGE), amplification and sequencing (MLST and *spa*) at least five times.

## Discussion

The BEC clone, first described in 1992 in Brazil, is a universally occurring multidrug-resistant linage, endemic in Brazilian hospitals and predominant among HA-MRSA in the country. This clone displays some characteristics such as enhanced ability to produce biofilm, to adhere to and invade epithelial airway cells that could provide a great capacity for worldwide spread. In this context, BEC is responsible for a large number of HA-MRSA infections in several South American countries and in other continents [10, 13, 28–30].

In this study, BEC was the most common MRSA clone observed, representing 61 % of MRSA isolates. BEC isolates are known to be dispersed throughout Brazil, with a major clonal type being responsible for 70–80 % of BEC strains [30, 31]. More recently, increased variability in PFGE patterns for BEC isolates in Brazil has been described [11]. Similarly, we observed extensive variability of PFGE patterns for BEC isolates, suggesting clonal divergence over time. This observation reinforces the report of [11] that these genetic changes may have some significance in a particular epidemiological scenario and might correspond to an important instrument of clonal divergence.

Few data have been published on the incidence of MRSA and MSSA infection in Northeast Brazil. In one study, BEC accounted for 70 % of MRSA strains from a university general hospital in Recife during 2002–2003, with 14 % of strains being USA800/PC -related [13]. USA800-related strains were also common in the present study. All MRSA isolates carrying SCC*mec*IV were considered to be related to USA800 and were the second most frequent MRSA isolates found in Recife. These isolates were in general more homogeneous than BEC isolates by PFGE analysis. We also found several MSSA isolates related to the USA800 clone.

Some authors have reported that type IV MRSA isolates from different lineages can carry specific virulence factors (as presence of *egc* locus, *pvl* and biofilm production) and/or resistance genes [3, 13, 31, 32]. Thus, the USA800 isolates seems to have specific characteristics that give the bacteria the ability to spread and promote their emergence as important pathogen in hospital settings worldwide. The isolates related to the peadiatric epidemic clone might conserve features that provide more homogeneity to these isolates which could afford their maintenance in hospital environment.

We describe one MRSA isolate (Sa81) related to the USA100/New York/Japan clone, which was isolated from the blood of a cardiology patient. Some studies in Brazil have reported the presence of strains related to the USA100, ST5 [13, 16, 33, 34]. A similar strain, USA100 with ST105, *spa* type t002, SCC*mec*II MRSA, was reported from Southeast [35], suggesting that this clone could be dispersed throughout the country.

We observed 30 MSSA isolates that carries the *mec*A gene, without *mec* complex amplification, referred as OS-MRSA. This phenomenon may occur by partial excision of SCC*mec* in multiresistant MRSA isolates or chromosomal integration of the cassette chromosome, resulting in MSSA isolates that contain SCC*mec* segments. Some studies have reported the presence of resistance determinants in MSSA isolates [7, 35]. An additional table file shows this in more detail (see Additional file 1). According to CLSI [20], staphylococcal isolates that carry the *mec*A gene or produce PBP2a must be reported as oxacillin resistant in hospital settings.

Results from Oliveira and de Lencastre [36] strongly suggest that the transcriptional control of the *mec*A gene is mediated either directly or indirectly by other yet unidentified determinants (other than system *mec*I-*mec*R1 from *mec* complex), resulting in the phenotypic expression of β-lactam resistance. Because testing for *mec*A and PBP2a is not a routine procedure performed by clinical microbiology laboratories in Brazil, clinical isolates could be misclassified as MSSA and have implications for treatment of patients with staphylococcal infection. It is important to emphasize that the OS-MRSA isolates observed appears to be genetically diverse. Some of them were related to epidemic clones as USA400, USA100/New York/Japan, USA800/PC and BEC. To our knowledge, this is the first study of prevalence of OS-MRSA infections in Brazil. Further research is required to better characterize the OS-MRSA isolates observed. Studies are needed to evaluate the epidemiology, virulence factors, dissemination and implications for clinical treatment of OS-MRSA in Brazil.

Deurenberg and Stobberingh [28] described that the transfer of the SCC*mec* to a MSSA lineage, with a common genetic background, possibly generated MRSA clones such as CC5, CC8, CC22, CC30 and CC45. Thus, either the acquisition or loss of SCC*mec* by MSSA and MRSA isolates respectively may provide resistant or susceptible isolates with similar genetic backgrounds, conserving virulence characteristics, which might persist simultaneously in the hospital environment and could be responsible for nosocomial infection.

We observed various MSSA isolates related to epidemic clones as the CA-MRSA USA400 and USA1100/SPW, clones that are becoming common in hospitals worldwide and are involved in many nosocomial infections [3]. In general, the CA-MRSA are considered more virulent than HA-MRSA due to the existence of several virulence factors [37, 38]. USA1100 (SPW) was described for the first time in Brazil in 2005 and continues to be described in hospitals in Brazil, as well as the CA-MRSA USA400, accounting for multiple types of medical problems [18, 39].

We also verified MSSA isolates related to the HA-MRSA USA800/PC, USA100/NY/J and USA600/BC. The Berlin clone has the capacity of causing high mortality in patients with MRSA bloodstream infections and has great capacity for global dissemination; however, infections caused by this clone remain scarce in Brazil [16, 17, 40, 41].

## Conclusion

In the present study, despite the number of isolates analysed, the clonal spread of MRSA and MSSA, including a high prevalence of oxacillin-susceptible *mec*A-positive *S. aureus*, was observed within several areas of the major hospital investigated (outpatient clinic, inpatient ward and ICU). We also verified closely related isolates between hospitals, suggesting a possible spread of these strains in the hospital environment that could be responsible for nosocomial infections. We documented the presence of several MRSA clones, as well as new MLST and *spa* types, that were responsible for severe infections in hospitalized patients. Some uncommon isolate genotypes were observed.

Our findings concerning the prevalence of OS-MRSA in clinical settings underscore the need of genotypic tests, in addition to phenotypic assays, to accurately identify MRSA. Moreover, our data alert to the necessity for development more effective strategies for epidemiological control of *S. aureus* in order to avoid an increase of hospital infections. Further studies are required to determine the degree of OS-MRSA spread throughout Brazil.

## Abbreviations

BEC, Brazilian epidemic clone; CA-MRSA, Community-associated Methicillin-resistant *S. aureus*; CC, clonal complex; CLSI, Clinical and Laboratory Standards Institute; HA-MRSA, Hospital-associated Methicillin-resistant *S. aureus*; ICU, intensive care unit; MGE, mobile genetic element; MIC, minimum inhibitory concentration; MLST, multilocus sequence type; MRSA, methicillin-resistant *S. aureus*; MSSA, methicillin-sensitive *S. aureus*; OS-MRSA, oxacillin-susceptible *mec*A-positive *S. aureus*; PBP2a, alternative penicillin-binding protein; PC, paediatric clone; PFGE, pulsed-field gel electrophoresis; SCC*mec*, staphylococcal cassette chromosome; *spa*CC, *spa* clonal complex; ST, sequence type; SWP, southwest pacific clone

## Additional file

Additional file 1: Reports of oxacillin-susceptible *mec*A-positive *Staphylococcus aureus* (OS-MRSA) worldwide. Table showing published reports of resistance determinants in MSSA isolates (OS-MRSA) around the world. (PDF 135 kb)
