# Supplementary material for: Clonal diversity and epidemiological characteristics of Staphylococcus aureus: high prevalence of oxacillin-susceptible mecA-positive Staphylococcus aureus (OS-MRSA) associated with clinical isolates in Brazil
Source: BMC Microbiol. 2016 Jun 21;16:115. doi: 10.1186/s12866-016-0733-4 (PMC4915036; doi:10.1186/s12866-016-0733-4)
Supplement: Additional file 1: — Reports of oxacillin-susceptible mecA-positive Staphylococcus aureus (OS-MRSA) worldwide. Table showing published reports of resistance determinants in MSSA isolates (OS-MRSA) around the world. (PDF 135 kb) [file 12866_2016_733_MOESM1_ESM.pdf]

Additional file 1. Reports of oxacillin-susceptible *mec*A-positive *Staphylococcus aureus* (OS-MRSA) worldwide.

**Country**	**Year of the report**	**Authors**
Germany	2003	Kampf et al.
Germany, Austria	2007	Witte et al.
Japan	2007	Hososaka et al.
USA	2008	Forbes et al.
Greece	2008	Ikonomidis et al.
United Kingdom	2010	Saeed, Dryden and Parnaby
USA	2010	Bearman et al.
Argentina	2011	Cuirolo et al.
USA	2012	Sharff et al.
Taiwan	2012	Chen et al.
China	2013	He et al.
Iran	2013	Jannati et al.
China	2014	Pu et al.
United Kingdom	2014	Saeed et al.
Africa	2015	Conceição et al.

References:

Kampf, G. et al. 2003. Inducibility and potential role of *mec*A-genepositive oxacillin-susceptible *Staphylococcus aureus* from colonized healthcare workers as a source for nosocomial infections. J. Hosp. Infect. 54:124-129.

Witte, W., B. Pasemann, and C. Cuny. 2007. Detection of low-level oxacillin resistance in *mec*A-positive *Staphylococcus aureus*. Clin. Microbiol. Infect. 13:408-412.

Hososaka, Y. et al. 2007. Characterization of oxacillin-susceptible *mec*A-positive Staphylococcus aureus: a new type of MRSA. J. Infect. Chemother. 13:79-86.

Forbes, B.A. et al. 2008. Unusual form of oxacillin resistance in methicillin-resistant *Staphylococcus aureus* clinical strains. Diagn. Microbiol. Infect. Dis. 61:387-395.

Ikonomidis, A. et al. 2008. In vitro and in vivo evaluations of oxacillin efficiency against *mec*A-positive oxacillin-susceptible *Staphylococcus aureus*. Antimicrob. Agents Chemother. 52:3905-3908.

Saeed K, Dryden M, Parnaby R. 2010. Oxacillin-susceptible MRSA, the emerging MRSA clone in the UK? J Hosp Infect.76:267–8.

Bearman, G.M. et al. 2010. Nasal carriage of inducible dormant and community-associated methicillin-resistant *Staphylococcus aureus* in an ambulatory population of predominantly university students. Int. J. Infect. Dis. 14(Suppl. 3):e18-e24.

Cuirolo A, Canigia LF, Gardella N, et al. 2011. Oxacillin- and cefoxitin- susceptible methicillin-resistant *Staphylococcus.* Int J Antimicrob Agents. 37:178–9.

Sharff, K. A., Monecke, S., Slaughter, S., Forrest, G., Pfeiffer, C., Ehricht, R., & Oethinger, M. 2012. Genotypic resistance testing creates new treatment challenges: two cases of oxacillin-susceptible methicillin-resistant *Staphylococcus aureus*. *Journal of clinical microbiology*, *50*(12): 4151-4153.

Chen, F.J. et al. 2012. mecA-positive *Staphylococcus aureus* with lowlevel oxacillin MIC in Taiwan. J. Clin. Microbiol. 50:1679-1683.

He, W. et al. 2013. Prevalence and molecular typing of oxacillinsusceptible *mec*A-positive *Staphylococcus aureus* from multiple hospitals in China. Diagn. Microbiol. Infect. Dis. 77:267-269.

Jannati, E. et al. 2013. Nasal colonization of *mec*A-positive, oxacillin susceptible, methicillin-resistant *Staphylococcus aureus* isolates among nursing staff in an Iranian teaching hospital. Am. J. Infect. Control 41:1122-1124.

Pu, W. et al. 2014. High incidence of oxacillin-susceptible *mec*A positive *Staphylococcus aureus* (OS-MRSA) associated with bovine mastitis in China. PLoS One 9:e88134.

Saeed, K. et al. 2014. Oxacillin-susceptible methicillin-resistant *Staphylococcus aureus* (OS-MRSA), a hidden resistant mechanism among clinically significant isolates in the Wessex region/UK. Infection 42:843-847.

Conceição, T., Coelho, C., de Lencastre, H., & Aires-de-Sousa, M. 2015. Frequent occurrence of oxacillin-susceptible *mec*A-positive *Staphylococcus aureus* (OS-MRSA) strains in two African countries. Journal of Antimicrobial Chemotherapy.
